# Regenerative strategies for ALS: stem cells and extracellular vesicles

**DOI:** 10.1186/s11671-026-04820-2

**Published:** 2026-08-17

**Authors:** Trisha Raghunathan, Balaji Parthasarathy, Prajnya Prabhu, Preetham Mahesh, Anandh Dhanushkodi

**Affiliations:** Manipal Institute of Regenerative Medicine, Manipal Academy of Higher Education, Madhav Nagar,, Manipal,, Karnataka 576104 India

**Keywords:** Amyotrophic lateral sclerosis, Adult stem cells, Neuroprotection, Exosomes, Neurodegeneration

## Abstract

Amyotrophic Lateral Sclerosis (ALS) is caused by progressive degeneration of upper and lower motor neurons. The disease is late onset, and to date, no early diagnosis is possible. Patients with ALS have a 5-year survival rate since diagnosis. Though recent studies highlighted the possible mechanisms of motor neuron degeneration in ALS, the treatment options are extremely limited. This underscores the urgent need to develop effective therapeutic strategies that can prolong patient survival and ultimately slow/halt ALS progression. Extracellular vesicles released from the degenerative milieu contribute to ALS propagation and progression by shuttling misfolded proteins, proinflammatory cytokines, and neurotoxins; thus, they could serve as a biomarker for diagnosis and prognosis. The advancement of stem cell-based therapies for neurodegenerative diseases and the evolving understanding of extracellular vesicles as potential biotherapeutics provide a ray of hope for millions of patients suffering from neurological disorders/neurodegenerative diseases like ALS.

**Graphical abstract:**

**Figure:**
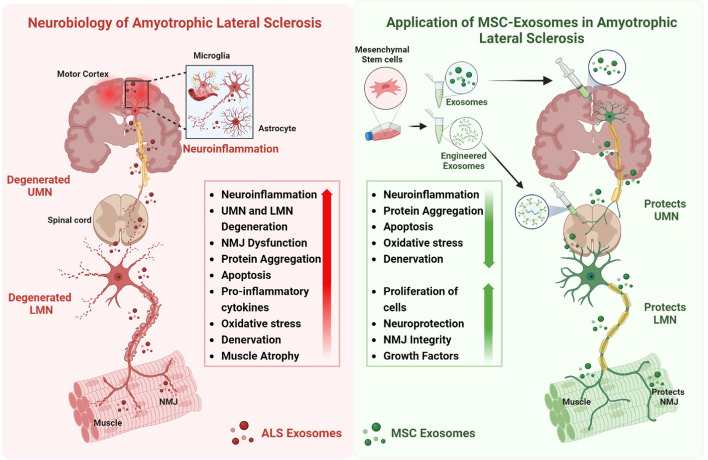

## Introduction

Amyotrophic Lateral Sclerosis (ALS) is a fatal, heterogeneous neurodegenerative disease characterised by the progressive degeneration of upper motor neurons (extending from the cortex to the brainstem or spinal cord) and lower motor neurons (originating in the spinal cord and innervating muscles). This degeneration leads to relentless muscle weakness, atrophy, and eventual paralysis [48, 69]. Weakness typically begins in the distal limb musculature before spreading proximally. ALS generally presents later in life, with most diagnoses occurring between 50 and 65 years of age. The disease is universally fatal, with a median survival of 2–5 years from symptom onset [63, 177].

Globally, ALS has an estimated prevalence of 4.42 per 100,000 individuals and an incidence of approximately 1.9 per 100,000 per year. Males are disproportionately affected, with prevalence rates of 5.96 compared to 3.90 in females [177]. Around 90% of ALS cases are sporadic, while the remaining 10% are familial, often associated with mutations in genes such as *SOD1*, *C9orf72*, *TDP-43*, and *FUS* [107]. The pathophysiology of ALS is multifactorial, involving mechanisms such as glutamate excitotoxicity, mitochondrial dysfunction, oxidative stress, neuroinflammation, and impaired proteostasis. This complexity makes the identification of a single effective therapeutic target particularly challenging [63, 88].

Despite decades of research, therapeutic options for ALS remain severely limited. Riluzole, a glutamate antagonist approved in 1995, and edaravone, an antioxidant, provide only modest symptomatic benefits and do not significantly alter disease progression [48]. A notable recent advancement was the FDA’s accelerated approval of tofersen (Qalsody) in April 2023, the first targeted gene therapy for ALS designed to reduce the production of toxic SOD1 protein in patients with *SOD1* mutations. Long-term follow-up data indicate that sustained treatment with tofersen slows disease progression, with approximately 25% of participants demonstrating functional stabilisation or improvement [115]. However, this therapy is applicable to fewer than 2% of ALS cases, leaving the majority of patients without disease-modifying treatment options.

At the same time, the withdrawal of sodium phenylbutyrate/taurursodiol (AMX0035/Relyvrio) following the failed Phase 3 PHOENIX trial highlights the fragility of the current therapeutic pipeline. The trial did not meet its primary endpoint, showing no significant difference in ALSFRS-R score changes between treatment and placebo groups at 48 weeks [129]. Consequently, supportive care including respiratory management, speech therapy, and nutritional support remains central to patient management [48].

In parallel, stem cells and extracellular vesicles (EVs) have emerged as promising areas of research in ALS, both as contributors to disease progression and as potential therapeutic tools. EVs are implicated in the prion-like spread of misfolded proteins, including TDP-43 and SOD1, which may drive the spatial and temporal progression of neurodegeneration in ALS [101]. Conversely, stem cell-derived EVs have gained attention as a cell-free therapeutic strategy. These vesicles may modulate neuroinflammation, reduce oxidative stress, and deliver neuroprotective molecules across the blood–brain barrier [43, 168]. Notably, a 2024 pilot clinical study reported that intravenous administration of human bone marrow-derived MSC-EVs was safe in ALS patients and showed early signs of disease stabilisation, supporting the need for larger, controlled trials [40]. This convergence of significant unmet clinical need and rapid advances in EV biology marks a critical juncture in ALS research. While previous reviews have examined either stem cell therapies or EV biology independently, no comprehensive synthesis has yet integrated both the role of EVs in disease propagation and their therapeutic potential. This review aims to address that gap by providing a unified and up—to-date perspective, particularly in light of recent FDA decisions and the accelerating pace of EV-based clinical research.

## Classification of ALS

Amyotrophic lateral sclerosis (ALS) is broadly classified into two forms: (a) sporadic or idiopathic ALS (sALS) and (b) familial ALS (fALS). The sporadic form accounts for approximately 90% of cases, while the remaining 10% are inherited [77]. Several genes have been implicated in both fALS and, to a lesser extent, sALS. Mutations in the *SOD1* gene, the first gene identified in association with ALS are responsible for approximately 20% of familial cases and around 3% of sporadic cases. 

More recent studies have identified additional genes involved in ALS pathogenesis, including *TARDBP* (TAR DNA-binding protein), *OPTN* (optineurin), *TBK1* (tank-binding kinase 1), *FUS* (fused in sarcoma), and *C9ORF72* (chromosome 9 open reading frame 72). Although these genes are primarily linked to hereditary ALS, they are also increasingly recognised as contributors to some cases of sALS. 

Current understanding of ALS pathogenesis is largely derived from studies of fALS, as the aetiology of sALS remains complex and multifactorial. While a positive family history can aid in identifying fALS, some cases classified as sporadic may in fact represent familial disease due to incomplete family history or reduced penetrance of pathogenic mutations [3].

## Mechanisms of neurodegeneration in ALS

Disease mechanisms in ALS are diverse at the physiological, molecular, cellular, genetic, and epigenetic levels. All these factors contribute to the disease's progression and severity, causing motor neurodegeneration and muscle loss in ALS patients [170]. In the subsequent sections, we summarize the various mechanisms involved in the neurodegeneration of motor neurons in ALS.

### Cortical hyperexcitability and glutamate excitotoxicity

Cortical hyperexcitability is a well-established and early pathophysiological feature of ALS, now recognised as a pre-symptomatic phenomenon that precedes clinical motor neuron degeneration and may serve as a biomarker for early disease detection [66, 176]. The “dying-forward hypothesis” proposes that neurodegeneration originates in the cortical motor areas, particularly involving Betz cells and propagates anterogradely via glutamate-mediated excitotoxicity to lower motor neurons, with cortical hyperexcitability acting as the primary driver of this cascade [68, 176]. Degeneration of inhibitory interneurons in the primary motor cortex plays a key role in this process, contributing significantly to cortical hyperexcitability and network dysfunction [66].

Clinically, cortical hyperexcitability manifests as muscle spasticity, hyperreflexia, and fasciculations. Neurophysiological studies have shown that patients with ALS exhibit significantly reduced short-interval intracortical inhibition, accompanied by increased short-latency intracortical facilitation. This imbalance between inhibitory and facilitatory circuits contributes to the functional deficits observed in ALS [96, 136]. Recent work further highlights that this hyperexcitability arises from a complex interplay between distinct inhibitory and excitatory interneuron populations within the motor cortex, providing potential targets for therapeutic intervention [136]. Additionally, cortical hyperexcitability has been linked to early deficits in theta–gamma phase amplitude coupling and reduced noradrenaline levels in the motor cortex, implicating noradrenergic signalling deficiency as a novel non-cell autonomous contributor to excitability dysregulation [148].

At the level of peripheral axons, ion channel dysfunction also contributes to hyperexcitability. Persistent sodium channel conductance, increased sodium influx, and reduced outward potassium conductance result in repetitive action potential firing, ultimately promoting neuronal injury and death [160].

Sustained neuronal hyperexcitability converges on excitotoxicity, a key mechanism of neuronal death driven by overstimulation of glutamate-gated ionotropic receptors, leading to excessive calcium influx into motor neurons. Elevated intracellular calcium disrupts mitochondrial membrane potential and function, induces oxidative and endoplasmic reticulum stress, and activates pro-apoptotic pathways, collectively driving neurodegeneration [6]. Under physiological conditions, glutamate levels are tightly regulated by astrocytic transporters EAAT1/GLAST and EAAT2/GLT-1. In ALS, downregulation of EAAT2/GLT-1 leads to pathological accumulation of glutamate in the synaptic cleft, thereby promoting excitotoxicity in both familial and sporadic forms of the disease [6, 50].

Excitotoxicity is further exacerbated by alterations in AMPA receptor composition. The GluA2 subunit normally renders AMPA receptors impermeable to calcium; however, this protective mechanism is compromised during ALS progression, resulting in increased calcium permeability. A key factor underlying this disruption is impaired Q/R site editing of GluA2 pre-mRNA by adenosine deaminase acting on RNA 2. In sporadic ALS, adenosine deaminase acting on RNA 2 expression is significantly reduced or absent in spinal motor neurons, leading to the formation of calcium-permeable AMPA receptors [6, 72]. This deficiency is closely linked to TDP-43 pathology. Recent studies further demonstrate that TDP-43 dysfunction induces mis-splicing of the *KCNQ2* potassium channel, disrupting an essential neuronal “braking” mechanism and triggering intrinsic hyperexcitability. This provides a direct mechanistic link between TDP-43 proteinopathy and the excitotoxic cascade in ALS [58, 82].

### Oxidative stress and mitochondrial dysfunction in the ALS neurons

In ALS motor neurons, pathological calcium influx driven by excitotoxicity disrupts mitochondrial membrane potential, impairs electron transport chain function, and promotes excessive generation of reactive oxygen species, establishing a self-reinforcing cycle of oxidative damage and mitochondrial failure [8, 135]. Mitochondrial dysfunction in ALS is characterised by impaired energy metabolism, dysregulated calcium homeostasis, disrupted mitochondrial dynamics including abnormal fission and fusion and defective mitophagy, all of which converge to accelerate motor neuron degeneration [5, 135].

Motor neurons are particularly vulnerable to this oxidative cascade due to their intrinsically limited calcium-buffering capacity and relatively weak antioxidant defences, rendering them more susceptible to calcium-mediated injury than other neuronal subtypes [8]. Elevated intracellular calcium further activates pro-apoptotic pathways, including calpain- and caspase-dependent cascades, which exacerbate mitochondrial damage and ultimately drive neuronal cell death [135].

In *C9orf72*-associated ALS models, mitochondrial morphological abnormalities, increased oxidative stress markers, and impaired mitophagy have been directly linked to locomotor decline. Notably, targeting the Keap1/Nrf2 antioxidant pathway has been shown to mitigate oxidative stress and improve motor function, highlighting a promising therapeutic strategy [7].

Importantly, reactive oxygen species generated within degenerating motor neurons do not act in isolation but also affect surrounding glial cells. Oxidative stress impairs astrocyte function and compromises their critical role in maintaining the integrity of the blood–brain barrier and blood–spinal cord barrier. Disruption of these barriers facilitates the entry of neurotoxic plasma components and immune cells into the central nervous system, thereby amplifying neuroinflammation and accelerating disease progression in ALS [90].

### Glial activation and neuroinflammation

Glial cells play a central role in maintaining central nervous system homeostasis, performing functions such as neurotransmitter synthesis and reuptake, metabolic support, potassium buffering, immune surveillance, and maintenance of the blood–brain barrier. In ALS, these essential functions are profoundly disrupted through glial activation, which accelerates motor neuron degeneration via non-cell autonomous mechanisms [74, 105].

Astrocytes, the most abundant glial cells in the CNS, are critical for maintaining glutamate homeostasis, ionic balance, and BBB integrity through their perivascular end-feet. Under pathological conditions in ALS, astrocytes undergo reactive astrogliosis, a remodelling process characterised by cellular hypertrophy, proliferation, loss of homeostatic function, and acquisition of a neurotoxic phenotype [35]. This reactive state is broadly categorised into two subtypes: the A1 neurotoxic and A2 neuroprotective phenotypes. A1 astrocytes upregulate complement cascade genes, secrete pro-inflammatory cytokines such as IL-1α, TNF-α, and IL-6, and contribute directly to neuronal and oligodendrocyte death. In contrast, A2 astrocytes promote neuronal survival through the release of anti-inflammatory and neurotrophic factors [74, 181]. In ALS, the A1 phenotype predominates, driven in part by activated microglia that release IL-1α, TNF-α, and C1q, thereby inducing the transformation of quiescent astrocytes into neurotoxic A1 cells [105]. Beyond cytokine secretion, reactive astrocytes exhibit metabolic and functional abnormalities, including lipid droplet accumulation particularly in cells expressing mutant TDP-43, disrupted lactate metabolism, impaired glutamate clearance due to downregulation of EAAT2/GLT-1, and the release of neurotoxic factors through an altered secretome. Collectively, these changes deprive motor neurons of essential metabolic and trophic support while exacerbating excitotoxicity and inflammation [105, 181].

Microglia, the resident immune cells of the CNS, are the principal mediators of neuroinflammation in ALS. Upon activation, they can adopt a pro-inflammatory M1-like phenotype, characterised by the production of reactive oxygen species (ROS), nitric oxide, and cytokines such as IL-1β, IL-6, and TNF-α, or an anti-inflammatory, neuroprotective M2-like phenotype associated with the release of IL-4, IL-10, and TGF-β [55, 75]. Although the M1/M2 classification is now considered an oversimplification given the identification of diverse microglial states such as disease-associated microglia and neurodegenerative microglia through multi-omics approaches, it remains a useful framework for understanding functional shifts of microglia during disease progression [124, 125]. In SOD1-G93A mouse models, early disease stages are marked by a predominance of M2-like microglial markers, suggesting an initial neuroprotective response aimed at preserving motor neuron viability. However, as the disease progresses, microglia shift towards a pro-inflammatory M1-like phenotype, which accelerates motor neuron degeneration and worsens clinical outcomes. This transition is influenced by the evolving microenvironment, including reactive astrocytes, ALS-associated protein aggregates, and infiltrating peripheral immune cells [55, 124, 125].

**Fig. 1:**
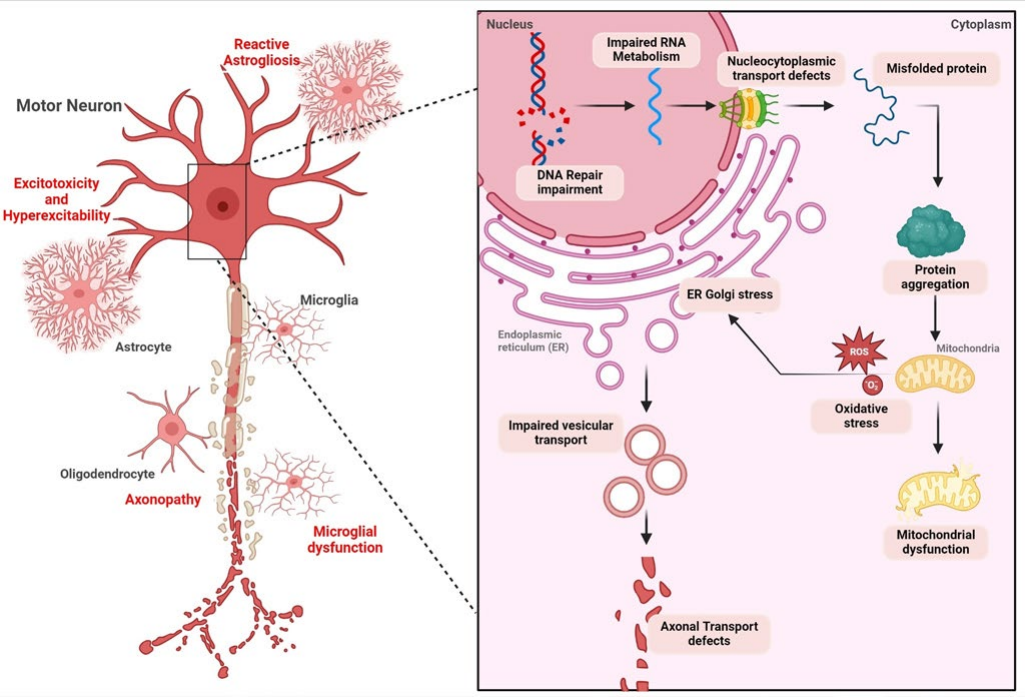
The Motor neurons in ALS undergo various pathogenic mechanisms. ALS is characterized by the progressive degeneration of upper and lower motor neurons, driven by both cell-autonomous and non-cell-autonomous mechanisms. The left panel illustrates the contribution of surrounding glial cells, including reactive astrocytes, activated microglia, and dysfunctional oligodendrocytes, which promote neuroinflammation, excitotoxicity, and neuronal hyperexcitability, thereby exacerbating motor neuron injury. The right panel depicts the major intracellular pathogenic pathways implicated in motor neuron degeneration. Genetic mutations and cellular stress lead to impaired RNA metabolism, defective nucleocytoplasmic transport, and compromised DNA repair. These disturbances promote endoplasmic reticulum and Golgi stress, resulting in protein misfolding and aggregation. Concurrently, oxidative stress and mitochondrial dysfunction impair cellular energy homeostasis and increase neuronal vulnerability. Defects in vesicular trafficking and axonal transport further disrupt the delivery of essential proteins and organelles along the axon, ultimately leading to synaptic failure and motor neuron death. The convergence of these interconnected pathways drives the progressive neurodegeneration that defines ALS

The importance of non-cell-autonomous mechanisms in ALS pathogenesis is further supported by studies in SOD1 mutant mouse models. Disease progression is significantly accelerated when mutant SOD1 is expressed in both neurons and glial cells, compared to expression in neurons alone. Conversely, selective deletion of mutant SOD1 from glial cells markedly slows disease progression, whereas reducing SOD1 levels exclusively in motor neurons has minimal effect. These findings highlight the critical role of dysfunctional glial cells particularly astrocytes and microglia, as key drivers of ALS pathogenesis [105, 124, 125]. Pathogenic mechanisms including cell and non-cell autonomous mechanisms in ALS is summarized in Fig. 1.

### Mutations associated with ALS disease progression

In fALS, multiple inheritance patterns have been described, including autosomal dominant, semi-dominant, autosomal recessive, and X-linked modes. These genetic forms involve both gain-of-function (GOF) and loss-of-function (LOF) mechanisms. GOF mutations arise either through increased gene expression, leading to excessive protein production, or through alterations in the coding sequence that produce structurally or functionally abnormal proteins. These mutations typically follow autosomal dominant or semi-dominant inheritance patterns, where a heterozygous mutation in a single allele is sufficient to disrupt cellular homeostasis. In contrast, LOF mutations are generally associated with recessive inheritance and result in reduced or absent production of functional protein. In some cases, LOF can also manifest through haploinsufficiency, where a single functional allele fails to produce adequate protein levels to maintain normal cellular function [163].

ALS exhibits marked genetic heterogeneity, with more than 40 genes identified as causative or disease-associated. Collectively, these account for approximately 10% of fALS cases, while the remaining 90% are classified as sporadic [62, 172]. Among these, four major genes *SOD1*, *C9orf72*, *TARDBP*, and *FUS* account for up to 70% of fALS cases in European populations [172].

Autosomal dominant mutations in *SOD1* lead to toxic protein misfolding, oxidative stress, mitochondrial dysfunction, and non-cell-autonomous neuronal death, often resulting in a predominantly lower motor neuron phenotype [114]. The *C9orf72* hexanucleotide repeat expansion disrupts RNA metabolism, nucleocytoplasmic transport, and autophagy through the accumulation of toxic dipeptide repeat proteins, and is frequently associated with ALS–frontotemporal dementia, characterised by cognitive and behavioural impairment [14, 116]. Mutations in *TARDBP* result in cytoplasmic aggregation of TDP-43, defective RNA processing, stress granule formation, and axonal dysfunction. Similarly, *FUS* mutations impair DNA repair, RNA metabolism, and axonal integrity, and are often associated with early disease onset and rapid progression [9, 117]. Beyond these major genes, additional genetic contributors affect overlapping cellular pathways. Mutations in *OPTN* and *TBK1* disrupt selective autophagy and neuroinflammatory signalling through impairment of the OPTN–TBK1 axis and NF-κB signalling [14]. *NEK1* mutations are associated with ciliary dysfunction, impaired DNA damage repair, and cell cycle dysregulation [124, 125]. Mutations in *UBQLN2* impair ubiquitin–proteasome and autophagy–lysosomal pathways, while *VCP* mutations promote the accumulation of TDP-43-positive inclusions, endoplasmic reticulum stress, and lysosomal dysfunction [14]. Cytoskeletal integrity and axonal transport are disrupted by mutations in *PFN1*, *TUBA4A*, *DCTN1*, and *C21ORF2*, the latter also affecting DNA repair and mitochondrial function in motor neurons [187]. RNA metabolism is further perturbed by mutations in *hnRNPA1*, *TIA1*, and *ATXN2*, which promote stress granule formation and TDP-43 proteinopathy. Additionally, *GRN* mutations impair lysosomal function and modulate neuroinflammation, while *ALS2* and *SPG11* mutations are associated with autosomal recessive, juvenile-onset upper motor neuron-predominant disease [22, 172].

Collectively, the genetic architecture of ALS converges on three major pathological axes: impaired proteostasis and protein clearance, dysregulated RNA metabolism, and disrupted cytoskeletal and axonal dynamics. These converging mechanisms underscore the multifactorial nature of motor neuron degeneration and highlight the need for genotype-specific therapeutic strategies [14, 172].

## Pharmacological intervention

Therapeutic options for ALS remain limited. Currently, two drugs named Riluzole and Edaravone are approved for managing disease progression. In addition to pharmacological treatment, supportive interventions such as respiratory care, speech therapy, nutritional support, and physical rehabilitation are essential for improving quality of life, although they do not alter the underlying disease course [77].

Riluzole, a glutamate antagonist, is thought to reduce excitotoxicity by inhibiting glutamate release and activity [79]. Clinical studies have demonstrated that Riluzole modestly prolongs survival in patients with ALS [30, 165]. Edaravone, an antioxidant, reduces oxidative stress and neuroinflammation and has shown efficacy in clinical trials and an oral formulation has been approved for use in the United States. Both Riluzole and edaravone are associated with modest increases in patient survival [81].

In recent years, the therapeutic landscape has evolved with the emergence of precision gene therapies, particularly for fALS. Currently, gene therapies targeting mutant genes in fALS are being developed using CRISPR/Cas9 genome editing and antisense oligonucleotides (ASOs) [19]. ASOs are short, single-stranded nucleic acid molecules that bind to RNA and modulate protein expression. Tofersen, an ASO targeting *SOD1* mRNA, received accelerated FDA approval in 2023 for approximately 2% of ALS patients carrying *SOD1* mutations [16, 29]. Although the Phase 3 VALOR trial did not meet its primary functional endpoint at 28 weeks, Tofersen significantly reduced plasma neurofilament light chain and cerebrospinal fluid SOD1 protein levels and long-term follow-up data indicate that approximately 25% of patients treated over three years experienced functional stabilisation or improvement in measures such as grip strength and respiratory function [16, 115].

Jacifusen (ION363/ulefnersen), an ASO targeting *FUS* mRNA, is currently being evaluated in the Phase 3 FUSION trial (NCT04768972) for FUS-associated ALS. Early findings suggest that jacifusen reduces toxic FUS protein accumulation in spinal cord tissue and may slow disease progression, with at least one case demonstrating notable functional recovery following early intervention [150]. For *C9orf72*-associated ALS, several ASO-based approaches targeting the G4C2 repeat expansion are under investigation, including WVE-004 (NCT04931862). However, another candidate, BIIB078 (tadnersen), was discontinued after failing to demonstrate clinical benefit [113].

Gene therapy strategies using adeno-associated viral (AAV) vectors are also being explored, particularly for *SOD1*-related ALS. Preclinical and early clinical studies have shown delayed disease onset, prolonged survival, and preservation of neuromuscular junction function. In a small clinical study, AAV-delivered microRNA therapy was administered to two patients with *SOD1*-ALS to reduce mutant SOD1 protein expression in the spinal cord. While reductions in toxic protein levels were observed, one patient developed meningoradiculitis, necessitating immunosuppressive treatment. These findings highlight both the therapeutic potential and current limitations of AAV-based approaches [120].

## Stem cell therapy in ALS

Stem cells are self-renewable and can differentiate into many cell subtypes. Stem cells can be classified into embryonic stem cells, adult stem cells and induced pluripotent stem cells. Embryonic stem cells are derived from the inner mass of blastocysts, and they can differentiate into three germ cell progenitors. Adult stem cells are derived from many tissues such as bone marrow, placenta, dental pulp, and can be differentiated into multiple cell types. Induced pluripotent stem cells can be reprogrammed into embryonic stem cells. The most used stem cells for disorders of the brain are neural stem cells (NSCs), mesenchymal stem cells (MSCs), and induced pluripotent stem cells (iPSCs) [19].

## Neural stem cells (NSCs)

Neural stem cells (NSCs) have the capacity to differentiate into both neuronal and glial lineages and secrete a range of neurotrophic factors that support neuronal survival and growth [152]. Preclinical studies have demonstrated promising therapeutic potential of NSCs in ALS models, particularly through neuroprotective and paracrine mechanisms. A consortium study involving eleven double-blind experiments reported that transplantation of undifferentiated nestin-positive NSCs into the lumbar spinal cord of SOD1-G93A rodents improved motor coordination and extended lifespan by approximately 10 days. Notably, only ~1% of transplanted NSCs differentiated into neurons, predominantly during the mid-symptomatic stage, with negligible differentiation observed at the end stage of disease. Correspondingly, functional recovery was evident only in the mid-symptomatic group. These findings suggest that the therapeutic benefits are unlikely due to direct neuronal replacement, but rather mediated by non-neuronal cells and paracrine effects. Supporting this, NSC-conditioned media was shown to contain neurotrophic factors such as brain-derived neurotrophic factor (BDNF), nerve growth factor (NGF), and glial cell line-derived neurotrophic factor (GDNF), which are known to promote motor neuron survival [164]. Further evidence for a trophic mechanism comes from studies using genetically modified NSCs engineered to secrete GDNF. When unilaterally transplanted into the spinal cord of SOD1-G93A rats, these cells provided significant neuroprotection to motor neurons. However, they did not improve neuromuscular junction (NMJ) innervation or ipsilateral motor function, highlighting limitations in functional recovery [159]. Additional studies have demonstrated robust engraftment and functional integration of NSCs. Xu et al. [178] reported long-term survival and approximately 70% differentiation of transplanted human NSCs into neural cells (β-III tubulin-positive) in the lumbar spinal cord (L4–L5) of SOD1-G93A rats. These cells formed synaptic connections with host α-motor neurons, with evidence of reciprocal innervation. NSC transplantation also increased BDNF and GDNF levels in the cerebrospinal fluid and spinal cord, contributing to delayed disease onset, motor neuron preservation, and improved motor function.

In a subsequent study, dual transplantation of human NSCs into the spinal cord of pre-symptomatic SOD1-G93A mice resulted in migration to both grey and white matter, neuronal differentiation, and synaptic integration with host motor neurons. This intervention delayed disease onset and extended survival by approximately 10 days [179]. Zalfa et al. [186] further evaluated NSC transplantation at different disease stages in SOD1-G93A rats. Transplanted cells primarily integrated into laminae VIII–IX of the anterior horn and migrated along the rostrocaudal axis, reaching both thoracic and sacral spinal cord segments. These cells expressed markers of proliferation and differentiation into astrocytes, oligodendrocyte precursor cells, and mature neurons. Kaplan–Meier survival analysis showed a significant extension of lifespan by 28 days in treated animals. Disease severity was reduced, particularly in late-stage treated groups, and motor function was preserved by approximately 50% even at end-stage disease. Additionally, NSC therapy reduced misfolded SOD1 accumulation, astrogliosis, and microglial activation, further supporting its neuroprotective potential. Encouraged by these preclinical findings, early-phase clinical trials have evaluated NSC transplantation in ALS patients. Phase I and II studies indicate that NSC therapy is feasible, safe, and well-tolerated, with no major adverse effects reported following transplantation [47, 61, 108, 109].

## Mesenchymal stem cells (MSCs)

Stem cell therapy has been widely investigated for the treatment of neurodegenerative diseases, including ALS. Early hypotheses suggested that transplanted stem cells could transdifferentiate into local neuronal phenotypes and replace lost neurons. However, more recent evidence indicates that their therapeutic effects are primarily mediated through paracrine mechanisms. Specifically, transplanted stem cells secrete a diverse array of growth factors, anti-inflammatory cytokines, and EVs, which collectively promote endogenous repair processes within the host tissue [110]. Mesenchymal stem cells have emerged as a preferred candidate for ALS therapy due to their immunomodulatory properties and the feasibility of autologous transplantation. MSCs can be isolated from various tissues, including bone marrow, adipose tissue, placenta, peripheral blood, umbilical cord, and dental pulp [137, 169]. Numerous preclinical studies have demonstrated that MSCs can slow disease progression in both in vitro and in vivo ALS models. Multiple routes of MSC administration have been explored, including intraspinal, intrathecal, intraventricular, intravenous, and intralumbar delivery. Among these, intraspinal transplantation appears to be the most effective, as it enhances motor neuron survival, reduces astrogliosis, and slows disease progression [36, 171]. For instance, intravenous administration of stromal MSCs in irradiated SOD1-G93A mice resulted in cell survival for over 20 weeks, delayed disease onset by 14 days, and extended lifespan by 18 days [189]. Similarly, intraparenchymal transplantation of human bone marrow-derived MSCs into the spinal cord delayed disease onset, preserved motor neurons, reduced astrogliosis and microglial activation, and improved motor function. Notably, these cells survived for more than 10 weeks without the need for immunosuppression [171]. Dose-dependent effects have also been observed. In one study, intrathecal administration of BM-MSCs in SOD1-G93A mice demonstrated that higher doses (1 × 10⁶ cells) significantly delayed motor dysfunction, preserved motor neurons, and improved survival. Transplanted MSCs were shown to migrate to the ventricular system, subarachnoid space, brain, and spinal cord [89]. Furthermore, genetically engineered MSCs expressing glucagon-like peptide−1 reduced neuroinflammation, oxidative stress, and neuronal loss, while improving motor function and survival [94]. Adipose-derived MSCs have also been investigated. Intravenous administration of murine AD-MSCs at the symptomatic stage reduced motor function decline, although no significant improvement in lifespan was observed. These cells survived for up to 135 days and distributed to the brain, spinal cord (both grey and white matter), spleen, and muscle tissues. Increased levels of GDNF and basic fibroblast growth factor in the spinal cord suggest that their neuroprotective effects are mediated through trophic support [106]. Other administration strategies have shown promising outcomes. Intracerebroventricular delivery of MSCs delayed disease onset, extended lifespan by approximately 24 days, and exerted anti-apoptotic effects via upregulation of neurotrophic factors such as NGF, BDNF, vascular endothelial growth factor, and insulin-like growth factor-1 [91]. Similarly, systemic administration of human amniotic MSCs improved motor function, delayed disease onset by 13–14 days, prolonged survival by 11 days, and reduced neuroinflammation, as evidenced by decreased astrogliosis and microglial activation [158]. Dental pulp stem cells represent another promising MSC source. Derived from the fibrous connective tissue within the tooth, DPSCs are highly proliferative and relatively easy to obtain compared to bone marrow-derived stem cells. They exhibit fibroblast-like morphology, express stemness markers such as SOX-2, OCT-4, vimentin, and nestin, and can differentiate into neuronal lineages [169]. DPSCs can be isolated from third molars, deciduous teeth, or supernumerary teeth, making them a readily accessible and versatile cell source. Their multipotent differentiation capacity including osteogenic, chondrogenic, adipogenic, and neurogenic lineages further enhances their therapeutic potential [119]. Increasingly, attention has shifted toward the use of stem cell-derived conditioned media (CM), which contains bioactive factors secreted by cells, including growth factors, cytokines, functional proteins, metabolites, and EVs. DPSC-derived CM is particularly rich in neurotrophic factors such as BDNF, NGF, GDNF, neurotrophin-3 (NT-3), VEGF, and IGF-1, all of which contribute to neuronal survival, neurite outgrowth, axonal guidance, and neurogenesis [147, 169]. In SOD1-G93A mouse models, daily administration of DPSC-CM during pre-symptomatic stages reduced neuromuscular junction denervation in the gastrocnemius muscle and prevented motor neuron loss in the ventral horn. Importantly, treatment extended lifespan, although no significant changes were observed in astrogliosis or microglial activation, suggesting a direct neuroprotective effect on motor neurons [173]. In another study, DPSC-CM improved motor neuron survival, with factors such as growth differentiation factor 15 and heparin-binding EGF-like growth factor identified as key contributors to neuroprotection. Notably, heparin-binding EGF-like growth factor was shown to protect SOD1-G93A neurons from nitric oxide-induced toxicity and cell death [184].

### Schwann-cell like and Schwann-cell based approaches

The therapeutic potential of Schwann cell-based and Schwann cell-like approaches in ALS warrants particular attention, given the central role of lower motor neuron axonal degeneration and neuromuscular junction denervation in disease progression. Schwann cells are the principal myelinating and supportive glial cells of the peripheral nervous system, playing a critical role in axonal regeneration. They secrete neurotrophic factors such as NGF, BDNF, GDNF, and NT-3, and provide structural guidance to regenerating axons through the formation of regenerative pathways known as Bands of Büngner following injury [4]. The progressive axonal degeneration and NMJ denervation observed in ALS share key pathological features with peripheral nerve injury, making Schwann cell-targeted strategies mechanistically relevant. However, the clinical application of primary autologous Schwann cells is limited by several factors, including the need for invasive nerve harvesting, limited in vitro expansion capacity, and associated donor site morbidity [71]. To overcome these challenges, recent studies have demonstrated that MSCs derived from sources such as bone marrow, adipose tissue, and tonsils can be transdifferentiated into Schwann cell-like cells using defined induction protocols involving neurotrophic factors and signalling pathway modulators [122, 151]. These MSC-derived Schwann cell-like cells express key Schwann cell markers, including S100β, p75NTR, GFAP, and myelin basic protein. Functionally, they recapitulate the neuroprotective properties of native Schwann cells by secreting neurotrophic factors, promoting axonal growth, and modulating the peripheral nerve microenvironment [71, 122]. Notably, tonsil-derived MSC-derived Schwann cell-like cells, when delivered in a fibrin glue scaffold to peripheral nerve injury sites in rodent models, significantly enhanced sciatic nerve regeneration, reduced NMJ atrophy, and delayed muscle denervation, key pathological features relevant to ALS progression [122].

## Human-induced pluripotent stem cells (hiPSCs)

Human induced pluripotent stem cells (hiPSCs) represent a powerful platform for investigating the cellular mechanisms underlying selective motor neuron degeneration in ALS, as well as for drug screening and the generation of spinal motor neurons for potential cell-based therapies [45]. Numerous hiPSC models of familial ALS have been developed for key genes, including *SOD1*, *C9ORF72*, *TARDBP*, and *FUS* [59]. For example, motor neurons derived from hiPSCs carrying the *SOD1*A4V mutation exhibit increased vulnerability due to elevated oxidative stress and endoplasmic reticulum stress. Correction of this mutation using zinc finger nuclease-mediated gene editing significantly improved motor neuron survival, highlighting the causal role of the mutation [93]. Similarly, *C9ORF72* hiPSC-derived motor neuron models have revealed multiple pathogenic mechanisms, including RNA toxicity, dipeptide repeat protein toxicity, loss of functional *C9ORF72* protein, stress granule formation associated with ER stress, protein aggregation, and excitotoxicity [44]. hiPSC models based on *TARDBP* mutations have further elucidated defects in nucleocytoplasmic transport, contributing to the mislocalisation and aggregation of TDP-43, a hallmark of ALS pathology [37]. Collectively, these findings demonstrate that hiPSC-based models provide critical insights into the molecular and cellular mechanisms driving ALS pathogenesis [59]. Beyond disease modelling, hiPSC-derived neurons and glial cells also hold promise as a source for cell-based therapies. Several preclinical studies have reported beneficial outcomes following transplantation of iPSC-derived neural or glial cells, including enhanced neuroprotection, preservation of motor function, and extended survival in ALS models [45, 51, 54, 95, 123].

Table 1 summarises key studies demonstrating the therapeutic potential of cell-based approaches in ALS models.

**Table 1:** Summary of applications of stem cells in preclinical models of ALS

Model	Treatment	Mode of administration	Results	Reference
SOD1-G93A mice	Stromal MSCs	Intravenous	delayed disease onset, progression, and decrease in motor neuron loss and longer lifespan observed	[189]
SOD1-G93A mice	Human BM-MSCs	Intraparenchymal	Delayed disease onset, protected motor neurons and enhanced motor performance; Reduced astrogliosis and microglial activation	[171]
SOD1-G93A mice	BM-MSCs	Intrathecal	Reduced motor dysfunction preserves motor neurons and improves longevity and survival of the mice	[89]
SOD1-G93A mice	Engineered Glucagon-like peptide 1 (GLP-1)- Stromal MSCs	Intracerebroventricular	Improved motor performance and prolonged lifespan Decreased neuroinflammation and oxidative stress and nNOS levels	[94]
SOD1- mutant transgenic mice	Murine Adipose-derived MSCs (AD-MSCs)	Intravenous	Increased motor neuron survival, high levels of GDNF and bFGF, and modulation of glial cells	[106]
SOD1-G93A mouse	Human amniotic MSCs (hAMSCs)	Intravenous	Enhanced motor function, decreased motor neuron loss, reduced neuroinflammation, and increased levels of TGF-β, HGF, PGE2 and IDO factors and increased survival of mice	[158]
SOD1-G93A mice model	Human NSCs	Dual transplantation through Intraspinal and intraparenchymal	improved hind-limb motor function, preserved neuromuscular function, increased lifespan and survival, and delayed disease onset and progression. High levels of BDNF, NGF, and GDNF were found	[164]
SOD1-G93A rat	Genetically modified NSCs with GDNF	Unilateral transplantation into the spinal cord	Protection of motor neurons and spinal cord neurons	[159]
SOD1-G93A rat	Human NSCs	Lumbar protuberance	Robust engraftment, long-term survival of NSCs and 70% differentiation of cells into neuronal cells forming synaptic connections with surrounding neurons. Increased levels of GDNF and BDNF in CSF and spinal cord. Delayed disease onset, decrease in motor neuron loss and preserved motor function	[178]
SOD1-G93A	Human NSCs	Lumbar and cervical ventral horns of the spinal cord	NSCs migrated to the grey and white matter of the spinal cord, where they differentiated into neuronal cells and formed synaptic connections with the existing motor neurons. Prolonged life span, slowed disease onset and progression	
SOD1-G93A	Human NSCs	Anterior horns of the lumbar spinal cord	Increased survival and differentiation of NSCs in the spinal cord. Delayed progression of symptoms and reduced motor neuron loss and aggregated SOD1 levels. Decreased astrogliosis and microglial activation	[186]
SOD1-G93A mice	Dental pulp stem cell conditioned medium (DPSC-CM)	Intraperitoneal	administration of DPSC-CM in the early pre-symptomatic stage reduces the NMJ denervation. Higher survival of motor neurons of the ventral horn when administered daily, with extended lifespan of the mice	[173]
SOD1-G93A	DPSC-CM		GDF15 and HB-EGF were found in DPSC-CM. Exogenous HB-EGF has been shown to help the SOD1-G93A neurons from NO-toxicity and death	[184]
NOD-SCID mice	HSP-NSCs and hiPS-lt-NES cells	transplantation	Cells differentiate preferentially into glial lineage in the injured spinal cord, forming synaptic connections with the host neurons	[54]
SOD1-G93A mice	hiPSC-GRNPs	Lumbar spinal cord	transient improvement of lower limb function resulted in improved clinical scores of lower limbs; and upregulated expressions of endogenous VEGF and other neurotrophic factors; can differentiate into astrocytes expressing glutamate transporter 1 (GLT1)	[95]
SOD1-G93A mice	hiPSC-derived ALDHhiSSCloVLA4+ NSCs	Intrathecal and systemic injection	improved neuromuscular function and survival, neuroprotection, and positive host-environment modifications	[123]
SOD1-G93A mice	Transplantation of NP-iPS cells	Transplantation	preserve motor behaviour, increased survival and the density of neurons in the ventral horn; induce changes in cytokines and growth factors in the injected areas affecting the metabolism of ECM molecules	[51]

### Recent clinical studies on stem cell therapy in ALS

The most clinically advanced MSC therapy for ALS is debamestrocel (NurOwn), consisting of autologous bone marrow-derived MSCs engineered to secrete elevated levels of neurotrophic factors such as BDNF, GDNF and HGF, prior to intrathecal administration [15]. Although a Phase 3 randomised controlled trial (National Library of Medicine, NCT03280056 [23]) did not meet its primary endpoint in the overall ALS cohort, a subgroup of patients with less advanced disease showed a statistically significant slowing of progression, indicating a potential effect that is now being explored in the Phase 3b trial targeting earlier-stage patients [41]. Adipose-derived MSCs have also entered clinical evaluation, where a Phase 2 clinical trial data from a multisite intrathecal ASC trial (NLM, NCT03268603 [155]) similarly demonstrated no significant overall functional benefit but identified a subset of participant’s disease progression reduced at least by 25% following treatment, reinforcing the concept of biomarker-stratified patient selection for future trials [156]. Umbilical cord MSCs have gained additional interest following preclinical studies demonstrating that UC-MSC-conditioned medium prolongs survival in mouse model of ALS, attenuates microglial activation and astrogliosis, and suppresses neuroinflammatory pathways, representing a potentially more accessible and scalable cell-free delivery approach [52, 161]. Lenzumestrocel (Neuronata-R), an autologous bone marrow-derived MSC, is currently undergoing Phase 3 evaluation in ALS (National Library of Medicine, NCT04745299), representing one of the most advanced MSC clinical programmes. Based on the Phase 1 and 2 clinical trials of Lenzumestrocel, the injections showed significant benefits lasting upto six months in the ALS patients [121]. Another Phase 1/2 randomised double-blind, placebo-controlled multicentre clinical trial evaluated three intravenous doses of autologous adipose-derived MSCs in forty ALS patients on riluzole treatment. While the study demonstrated a favourable safety profile across all dose groups, functional efficacy measured by ALSFRS-R improvement was modest and did not reach statistical significance in the overall study, highlighting the challenge of patient heterogeneity in ALS cell therapy trials [2].

Furthermore, NSCs and glial progenitor cells represent another important avenue in the ALS therapeutic window. The NSI-566RSC human fetal spinal cord-derived NSCs were the first FDA-approved clinical trial for stem cell therapy in ALS, and subsequent Phase 1/2 studies demonstrated intraspinal injection safety at escalating doses, though functional improvements were modest [152]. More recently, novel immunomodulatory cell approaches have entered clinical development. CKO803, an allogenic umbilical cord blood-derived regulatory T-cell therapy, is under Phase 1 evaluation in the REGALS trial (National Library of Medicine, NCT05695521 [28]), leveraging CXCR3/CXCL10 signalling to engage with inflamed microglia in the ALS spinal cord. Notably the DSMB cleared progression to a second dose cohort following positive safety outcomes in 2024. RAPA-501, an autologous hybrid T-regulatory/Th2 T-stem cell therapy (NLM, NCT04220190 [144]), has entered Phase 2/3 evaluation in 2025, targeting immune dysregulation as a driver of motor neuron degeneration. NP001, an autologous regulatory T-cell administration being evaluated in Phase 1 trials (NLM, NCT06671236 [127]), further reflects the growing recognition that immunomodulation through T cell based approached represents a distinct band priming therapeutic axis in ALS. Collectively, as of December 2024, over 115 clinical trials worldwide have been approved to test 83 human pluripotent and stem cell derived products across various indications, with CNS diseases including ALS representing a major focus, and more than 1200 patients have been dosed with such therapies without generalised safety concerns [92]. Furthermore, Patient derived iPSCs carrying ALS linked mutations in SOD1, C9orf72, TARDBP and FUS have been successfully differentiated into motor neurons that recapitulate disease-specific pathological features including TDP-43 mislocalisation, axonal transport defects, and hyperexcitability, making them invaluable tools for disease modelling and drug screening [102, 128]. The FDA cleared XS-228, an allogenic iPSC derived motor neuron precursor therapy, to enter Phase 1 trials, following early phase studies demonstrating safety and preliminary signals of disease stabilisation. Concurrently, a separate iPSC-derived motor neuron precursor (National Library of Medicine, NCT06765564 [149]) has been started, offering advantage of enabling allogenic manufacturing from healthy donors, providing scalable and standardised cell products that overcome the time and cost limitations of autologous cell therapies [92, 102].

Beyond MSCs, iPSCs and adipose/Schwann cell-like approaches, the ALS cell therapy landscape includes several other distinct and important stem and progenitor cell types, each targeting different aspects of the disease’s pathophysiology. Olfactory Ensheathing Cells (OECs) represent one of the most biologically unique cell types investigated for ALS and motor neuron disorders. OECs are specialised glial cells that reside at the interface of PNS ad CNS, specifically within the olfactory mucosa and the olfactory bulb, where they support the lifelong regenerative capacity of olfactory receptor neurons. This dual CNS/PNS identity confers upon OECs a remarkable ability to promote axonal regeneration through secretion of multiple neurotrophic factors (GDNF, BDNF, NGF, VEGF), direct myelination of axons, matrix metalloproteinase modulation, and potent immunomodulatory activity [73, 100]. In early clinical studies, OEC transplantation in ALS patients via intrathecal delivery demonstrated acceptable safety and some signals of disease stabilisation, though functional improvements were modest and inconsistent [188]. Furthermore, characterisation of the human OEC secretome has identified 709 proteins including profilin-1, peroxiredoxin-1, and glutathione-s-transferase, with established neuroprotective and antioxidant relevance to ALS pathology, raising the possibility of OEC secretome based cell free therapeutic approaches as a safer and more scalable alternative to direct cell transplantation [73].

Glial progenitor cells (GPCs) have been pursued for their capacity to differentiate into motor neuron supportive astrocytes and oligodendrocytes, addressing the non-cell autonomous glial contributions to motor neuron death in ALS. A landmark Phase 1/2a clinical trial, evaluated CNS10-NPC-GDNF human progenitor cells engineered to expresses glial cell derived neurotrophic factor, transplanted directly into the lumbar spinal cord of 18 ALS patients (National Library of Medicine, NCT02943850), met its primary safety endpoint at one year with no negative side effects on motor function in the treated limb, and post mortem analysis of 13 deceased participants confirmed graft survival and sustained GDNF production for over three years [10, 92]. Recently, Human Embryonic Stem cell—Derived Astrocytes have also entered clinical evaluation for ALS. AstroRx, and hESC derived astrocyte, was administered intrathecally to ten ALS patients in a Phase 1/2a trial at two dose levels. While the treatment was well tolerated and the first three months post transplantation showed a slowing of disease progression rate, no significant functional improvement above baseline was maintained at the 12 month follow up reinforcing the challenge of sustaining early signs of neuroprotection in rapidly progressive ALS [92].

## Extracellular vesicles

Extracellular vesicles (EVs), once considered mere cellular debris, have gained significant attention in recent years due to their crucial role in intercellular communication [118]. EVs are secreted by a wide range of cell types, including epithelial cells, endothelial cells, neurons, mesenchymal stem cells (MSCs), dendritic cells, T and B lymphocytes, platelets, mast cells, and cancer cells [145]. Traditionally, EVs are classified into three major subtypes based on their biogenesis: exosomes, microvesicles, and apoptotic bodies [145].

Exosomes are nanosized vesicles, typically 30–150 nm in diameter, that play key roles in intercellular communication, disease propagation, and cellular protection [118]. One of the major challenges in treating central nervous system (CNS) disorders is the limited ability of therapeutic agents to cross the blood–brain barrier (BBB). Due to their small size and intrinsic ability to traverse the BBB, exosomes are considered promising vehicles for delivering biotherapeutic molecules to the CNS [80]. Recipient cells internalise exosomes via endocytosis, ligand–receptor interactions, or direct membrane fusion, leading to the release of their cargo, including proteins and RNAs such as microRNAs (miRNAs) and small interfering RNAs (siRNAs), which can modulate cellular function [152].

**Fig. 2:**
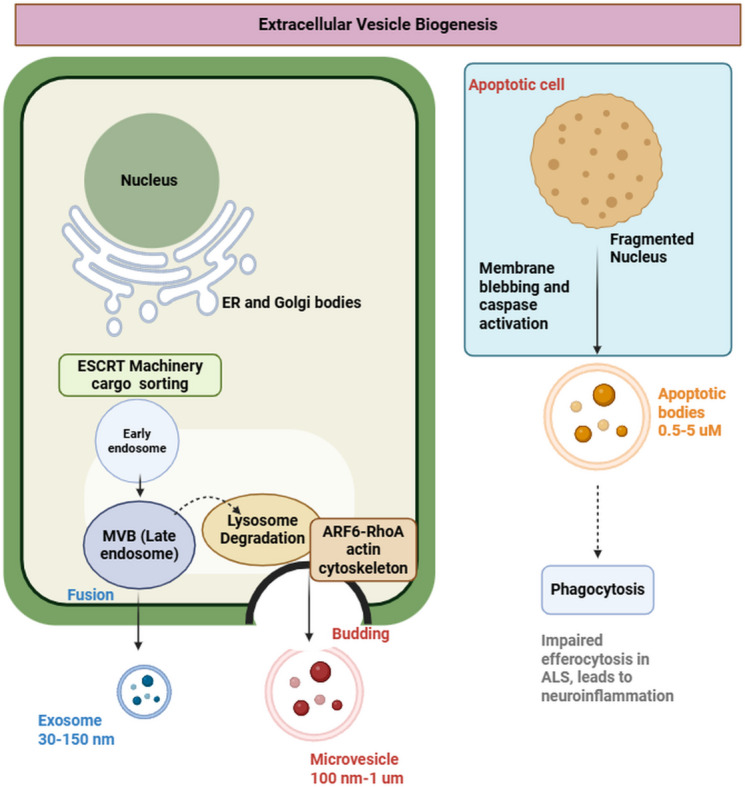
Graphical representation of the biogenesis of extracellular vesicles. Exosomes are generated through the endolysosomal pathway and subsequently released into the extracellular space. They contain a diverse range of cellular components, including proteins, lipids, nucleic acids, enzymes, growth factors, and chaperones. Microvesicles are formed by outward budding of the plasma membrane, a process regulated by ARF1, ARF6, Rab22a, and RhoA. This process involves activation of contractile machinery, cargo sorting, and cytoskeletal rearrangements. Apoptotic bodies are produced during programmed cell death, wherein cells undergo fragmentation and encapsulate intracellular components within large vesicles. These apoptotic bodies are subsequently cleared by phagocytes

Exosomes can be isolated using various techniques, including ultracentrifugation, differential ultracentrifugation, ultrafiltration, hydrostatic dialysis, size-exclusion chromatography, precipitation methods, and affinity-based approaches. Their isolation is generally based on properties such as size, density, solubility, and aggregation. Characterisation methods include electron microscopy or cryo-electron microscopy, as well as nanoparticle tracking analysis, which measures size distribution based on Brownian motion [85, 145]. Depending on their cellular origin, exosomes express a range of surface markers and proteins, including major histocompatibility complex molecules (MHC I and II), CD86, integrins, immunoglobulins, cell surface peptidases, tetraspanins, heat shock proteins, cytoskeletal proteins, and metabolic enzymes such as enolase 1 and thioredoxin peroxidase. These markers are widely used for exosome identification and characterisation [17, 67, 153, 166]. The biogenesis of exosomes is illustrated in Fig. 2.

Microvesicles are another class of EVs, typically ranging from 100 to 1000 nm in diameter, and are formed through direct outward budding of the plasma membrane. Their formation is regulated by intracellular calcium levels and cytoskeletal dynamics [27, 132]. Calcium influx alters membrane phospholipid composition, facilitating vesicle formation. Microvesicles are enriched in phosphatidylserine, CD40, cholesterol, ceramides, and sphingomyelins, and carry diverse cargo, including proteins, miRNAs, and mRNAs. Their biogenesis involves key regulatory proteins such as ARF1, ARF6, Rab22a, and RhoA, which coordinate membrane remodelling, cargo sorting, and cytoskeletal rearrangement. These processes contribute to their distinct composition, release dynamics, and functional roles in both physiological and pathological contexts [35].

Apoptotic bodies are large vesicles (500 nm to 5 µm) formed during programmed cell death. They arise from cellular fragmentation, encapsulating nuclear components, fragmented chromatin, and organelles [1]. Their formation can be triggered via intrinsic or extrinsic apoptotic pathways. The intrinsic pathway is initiated by cellular stress, leading to mitochondrial membrane permeabilisation and the release of cytochrome c and apoptosis-inducing factors. The extrinsic pathway involves activation of death receptors, such as Fas, through binding of Fas ligand, which recruits Fas-associated death domain proteins [33]. Under normal physiological conditions, apoptotic bodies are rapidly cleared by phagocytes through efferocytosis, a process that typically suppresses inflammatory responses [24, 174].

## Extracellular vesicles in ALS propagation and progression

In approximately 97% of ALS patients, misfolding and aggregation of TDP-43 have been reported [104]. Similarly, intracellular protein inclusions also arise from the aggregation of SOD1, FUS, and dipeptide repeat proteins generated from the *C9ORF72* intronic hexanucleotide repeat expansion [38]. These protein aggregates are highly toxic to both neurons and glial cells. A growing body of evidence suggests that misfolded proteins can propagate between cells in a prion-like manner, contributing to disease spread [83, 111]. Given the fundamental role of EVs in transporting biomolecules between cells, their involvement in disease propagation is increasingly recognised, particularly in cancer and neurodegenerative disorders. EVs can shuttle misfolded proteins, dysfunctional RNAs, and toxic molecules to neighbouring cells or distribute them systemically, thereby affecting distant regions beyond the initial site of pathology [97, 154]. Analogous to the spread of prion diseases via EVs [70], recent studies have demonstrated that EVs can mediate the transmission of mutant SOD1, FUS, TDP-43, and dipeptide repeat proteins associated with *C9ORF72*-linked ALS/FTD [38, 64, 126, 175]. Once internalised by recipient cells, these misfolded proteins can induce further protein aggregation, trigger neuroinflammation, impair mitochondrial function, deplete ATP levels, increase oxidative stress, and activate cell death pathways. Collectively, these processes contribute to the progressive spread of neurodegeneration across both proximal and distal regions of the nervous system. Several in vitro studies have demonstrated the transfer of mutant proteins via exosomes to neurons, astrocytes, and microglia, ultimately leading to cellular dysfunction and death [13, 64, 138, 175]. Notably, Feiler et al. [46] identified TDP-43 oligomers within EVs and showed that recipient cells preferentially internalise exosome-associated TDP-43 over its free form, highlighting the efficiency of EV-mediated transmission. In transgenic ALS mouse models, circulating EVs have been found to contain elevated levels of pro-inflammatory cytokines such as IL-1β, IL-6, and IFN-γ, suggesting a role in amplifying neuroinflammation within the central nervous system [167]. In addition to protein transfer, exosomes can also transport dysregulated or pathogenic microRNAs from affected to healthy cells, thereby altering gene expression and cellular function in recipient cells and further contributing to disease progression [167].

Microvesicles derived from ALS patients are enriched in SOD1, TDP-43, phosphorylated TDP-43, and FUS proteins compared to those from healthy controls. Notably, SOD1 is generally more concentrated in exosomes than in microvesicles, whereas TDP-43 and FUS levels tend to be slightly higher in microvesicles than in exosomes [154]. This differential distribution across EV subtypes has important implications for identifying which vesicle populations carry the greatest pathological burden and may therefore represent more effective therapeutic targets. In addition, EVs released from skeletal muscle have been shown to propagate toxic factors to other components of the neuromuscular junction, contributing to its destabilisation and the progressive decline in motor function. Furthermore, miRNAs packaged within EVs play a critical role in regulating muscle homeostasis, plasticity, and myogenesis under both physiological and pathological conditions. Dysregulation of these EV-associated miRNAs can therefore impair neuromuscular junction integrity and exacerbate disease progression in ALS [26].

## Extracellular vesicles serve as biomarkers in ALS

The lack of reliable biomarkers in ALS presents a major challenge for determining disease stage, often delaying diagnosis and the initiation of therapeutic interventions. EVs, due to their ability to cross biological barriers such as the blood–brain barrier and blood–spinal cord barrier, are readily detectable in accessible biological fluids including blood, cerebrospinal fluid, urine, and saliva. This accessibility has positioned EVs, particularly exosomes as promising candidates for biomarker discovery in ALS. Recent studies have reported elevated levels of TDP-43, SOD1, and FUS in plasma-derived exosomes from ALS patients [31, 154]. Additionally, ubiquitin-like modifying activating enzyme 1, gelsolin, and clusterin were found to be significantly upregulated in both plasma and CSF-derived exosomes from ALS patients carrying *C9ORF72* mutations [142]. In CSF-derived exosomes, increased levels of TDP-43, versican, programmed cell death 6 interacting protein, and neuro-ocular syndrome proteins 1 and 2 have also been reported [49, 112, 134]. Cell-type-specific EVs provide additional insights into disease progression. For instance, astrocyte-derived exosomes exhibit upregulation of cytokines, particularly IL-6, which correlates with scores on the revised ALS Functional Rating Scale [34]. Similarly, exosomal coronin-1α levels have been shown to positively correlate with disease progression [190]. In addition to protein biomarkers, non-coding RNAs, especially miRNAs within EVs are emerging as valuable diagnostic tools. Banack et al. [11] reported increased levels of miR-146a-5p, miR-199a-3p, miR-151a-3p, miR-151a-5p, and miR-199a-5p, alongside decreased levels of miR-4454, miR-10b-5p, and miR-29b-3p in plasma EVs from ALS patients. Exosomal miR-124 levels were found to be elevated in SOD1-G93A spinal cord neurons and positively correlated with disease severity [87]. Additional studies have reported increased miR-3919—3p and decreased miR-4454 and miR-4286 in ALS-derived EVs [32]. Furthermore, Yelick et al. [183] demonstrated a significant correlation between exosomal miR-124—3p levels and ALS Functional Rating scores. Despite these promising findings, it is important to note that alterations in EV-associated proteins and miRNAs are not specific to ALS and may also occur in other neurological disorders. Therefore, caution must be exercised when interpreting EV-based biomarkers. Rather than relying on single markers, the development of multiplex biomarker panels incorporating both protein and RNA signatures is likely to provide greater diagnostic accuracy for identifying disease onset and monitoring progression in ALS.

Sproviero et al. (2019) investigated plasma-derived microvesicles (MVs) in 40 ALS patients, compared with 28 patients with Alzheimer’s disease and 36 healthy controls. They reported that leukocyte-derived microvesicles (LMVs), identified by CD45⁺/Annexin V⁺ immunophenotyping, were significantly elevated in ALS patients relative to both comparison groups. Importantly, LMV levels showed a statistically significant correlation with disease progression, as measured by regression rate at the final clinical assessment [154]. Supporting these findings, another study reported similarly elevated levels of LMVs (CD45⁺ MVs) in the cerebrospinal fluid of ALS patients, reinforcing the biological relevance of this marker across both central and peripheral biofluids [12]. Collectively, these observations position LMVs as promising prognostic biomarkers capable of distinguishing ALS from other neurodegenerative disorders [154]. A notable recent development is the identification of astrocyte-derived EVs expressing GLAST (glutamate aspartate transporter) as a disease-relevant biomarker population in ALS. Raineri et al., [143] demonstrated that GLAST⁺ EV levels were significantly elevated in ALS patients compared to healthy controls. Emerging evidence further suggests that apoptotic bodies released from degenerating neurons may contribute to disease propagation. These vesicles can transfer nuclear components, including TDP-43 fragments, to neighbouring microglia and astrocytes, potentially acting as secondary vectors for the spread of proteinopathy. In addition, they may activate innate immune responses by recognising damage-associated molecular patterns, thereby amplifying neuroinflammation [1, 27].

## Extracellular vesicles as a biotherapeutic in ALS

Mesenchymal stem cell-derived exosomes retain many of the functional properties of their parental cells, including immunomodulatory and regenerative capabilities. Mesenchymal stem cells are known to secrete relatively high quantities of exosomes compared to other cell types. Importantly, MSCs can be sourced from diverse tissues such as bone marrow, umbilical cord, placenta, adipose tissue, and dental pulp, enabling the generation of tissue-specific exosomes that may be tailored for organ or disease specific therapeutic applications.

The therapeutic potential of MSC-derived exosomes has been demonstrated in both in vitro and in vivo models of ALS. Using the NSC-34 motor neuron-like cell line expressing mutant SOD1 to model familial ALS, Bonafede et al. [20] showed that exosomes derived from adipose-derived stem cells protected motor neurons against oxidative stress. This neuroprotective effect was attributed, in part, to exosomal miRNAs such as miR-21, miR-222, and let-7a, which are known to inhibit apoptosis and promote cell survival [20]. Consistent with these findings, Lee et al. [98] reported that adipose-derived MSC exosomes reduced SOD1 aggregation and restored mitochondrial function. Further supporting their therapeutic relevance, Gschwendtberger et al. [65] demonstrated that MSC-derived exosomes improved survival of primary motor neurons from SOD1-G93A mice subjected to staurosporine-induced neurotoxicity. This effect was mediated by the presence of ciliary neurotrophic factor within the exosomes, which activated the PI3K/AKT signalling pathway and promoted axonal sprouting. Additionally, MSC-derived exosomes have been shown to contain functional antioxidant enzymes, including catalase, superoxide dismutases, glutathione peroxidase, and thioredoxin reductase, which contribute to the mitigation of oxidative stress [57, 182]. Exosomal miRNAs further support redox homeostasis by modulating oxidative stress-related pathways in recipient cells [103, 130]. Given that oxidative stress is a central mechanism of motor neuron degeneration in ALS, the high levels of antioxidant components within MSC-EVs are particularly relevant [20, 25, 65].

In vivo studies further highlight their therapeutic promise. Repeated intranasal administration of adipose-derived MSC exosomes in SOD1-G93A mice, from disease onset to end stage, prevented motor neuron degeneration, reduced neuroinflammation, and preserved motor function. This suggests that intranasal delivery of MSC-EVs may represent a non-invasive and effective therapeutic strategy for ALS [21, 42, 168]. However, the therapeutic efficacy of exosomes appeared to decline at later stages of disease, indicating that dose optimisation and timing of administration require further investigation. In addition to their neuroprotective effects, MSC-derived exosomes may also address vascular dysfunction in ALS. Vascular pathology in ALS involves endothelial degeneration, impaired transport mechanisms, and astrocyte dysfunction, leading to blood–brain barrier disruption. Exosomes derived from bone marrow endothelial cells have been shown to prevent endothelial damage induced by plasma from SOD1-G93A mice [56]. Furthermore, proteomic analyses of MSC-derived exosomes have identified proteins involved in cell survival, angiogenesis, neurogenesis, cell adhesion, cytoskeletal organisation, and extracellular matrix–receptor interactions [18], suggesting that these vesicles promote neural repair through multiple complementary pathways.

An emerging therapeutic application more prominently associated with microvesicles than exosomes is the direct transfer of functional mitochondria to recipient cells. In addition to their immunomodulatory potential of EVs [174], microvesicles derived from MSCs have been shown to exert superior mitochondrial restorative effects compared to small EVs, with the capacity to transfer intact, functional mitochondria to metabolically impaired cells, thereby rescuing mitochondrial dysfunction [139]. Apoptotic bodies, one of the kind of EVs also emerging as potential therapeutic mediators with their roles in tissue regeneration, immunomodulation, and drug delivery [185]. Their immunomodulatory function is mediated through several interconnected mechanisms. Notably, phosphatidylserine residues exposed on the outer membrane of apoptotic bodies act as “eat-me” signals, engaging phosphatidylserine receptors such as TIM-4, MFG-E8, and BAI1 on macrophages and dendritic cells. This interaction promotes efferocytosis, which suppresses pro-inflammatory cytokine production while enhancing the release of anti-inflammatory mediators such as IL-10 and TGF-β [35]. In the context of ALS, a particularly relevant mechanism is the ability of apoptotic bodies to attenuate Toll-like receptor 5 (TLR5)/NF-κB-mediated inflammatory signalling in microglia. This pathway is also activated by misfolded SOD1 and TDP-43 aggregates, which drive glial mediated neurotoxicity. By modulating this pathway, apoptotic bodies may help counteract one of the key inflammatory cascades contributing to ALS progression [27, 86].

## Exosome for drug delivery into CNS

Mesenchymal stem cell-derived exosomes (MSC-EVs) possess several advantages as therapeutic delivery vehicles, including low immunogenicity, high biocompatibility, and the ability to selectively target inflamed tissues. Compared to synthetic delivery systems such as liposomes, nanocapsules, and dendrimers, exosomes exhibit greater stability due to their native lipid bilayer structure, reduced toxicity, and minimal induction of immune responses [99, 141]. These properties make exosome-based therapeutics particularly attractive for neurodegenerative diseases, where efficient crossing of biological barriers and targeted delivery are critical [67]. Indeed, studies have demonstrated the successful delivery of RNA cargo into the central nervous system (CNS) via exosomes [84].

Exosomes can be engineered to carry therapeutic molecules, including microRNAs (miRNAs) and small interfering RNAs (siRNAs). One common strategy involves modifying parental cells using viral vectors or plasmid transfection, enabling the incorporation of desired biomolecules into secreted exosomes and enhancing their functional specificity [99]. Alternatively, the exosomal cargo can be modulated through preconditioning of the parent cells under specific in vitro conditions. For example, exposure of MSCs to cerebrospinal fluid (CSF), plasma from ALS patients, or pro-inflammatory stimuli can alter the molecular composition of released EVs. Giunti et al. [60] demonstrated that interferon-γ preconditioning of bone marrow-derived MSCs enriched EVs with miR-466q and miR-467f, which reduced tumour necrosis factor and IL-1β levels and inhibited microglial activation, highlighting the potential for generating disease-specific therapeutic exosomes.

**Fig. 3:**
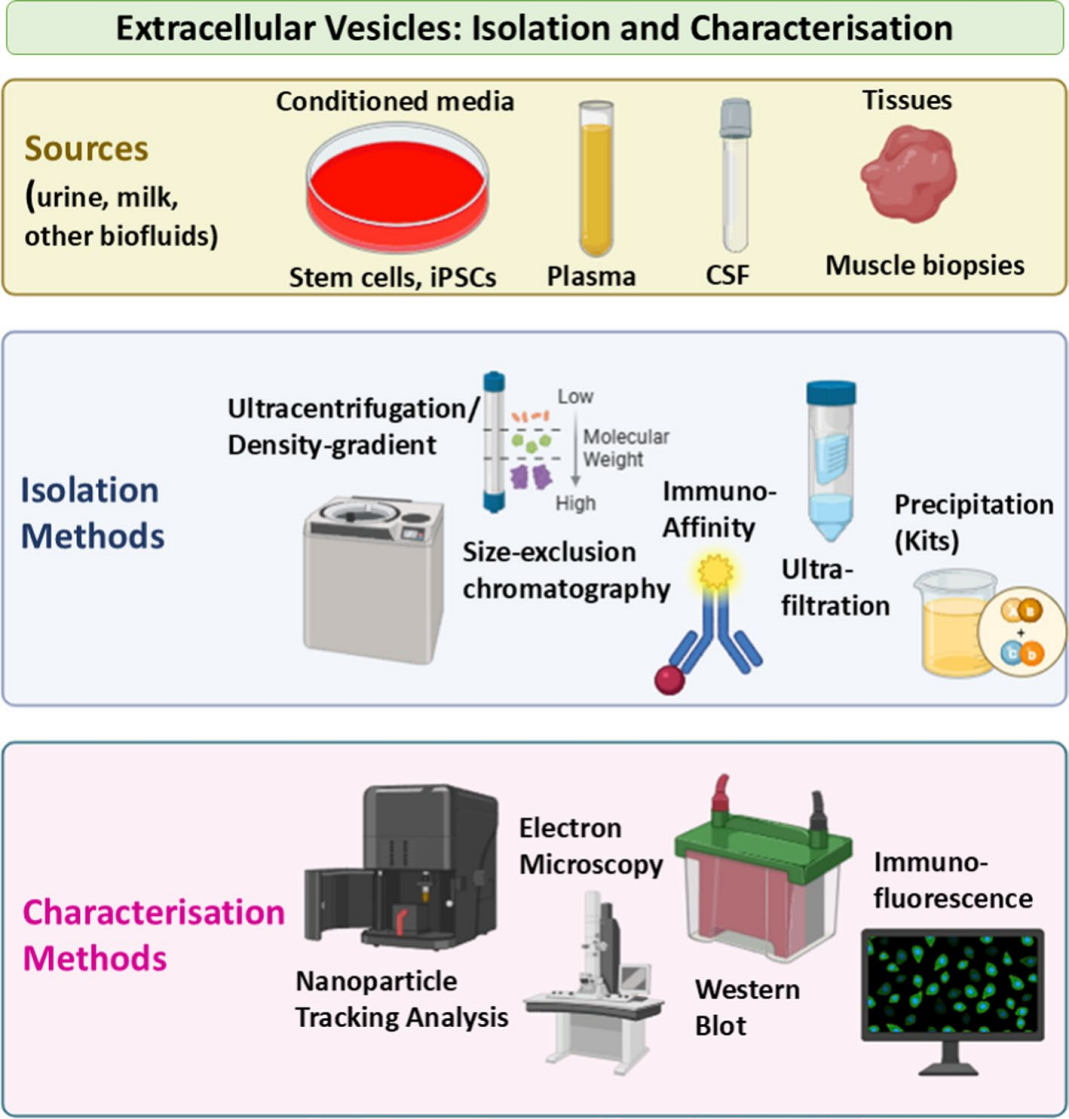
Graphical representation of the sources, isolation and characterisation of extracellular vesicles. EVs can be derived from a range of biological sources, including conditioned media from stem cells and iPSCs, biofluids such as plasma, CSF, urine and milk, as well as tissue homogenates and muscle biopsies, each offering different advantages depending on the experimental or clinical context. The choice of isolation method determines the EV yield, purity and downstream applicability. Ultracentrifugation and density-gradient ultracentrifugation exploit differences in vesicle size and buoyant density to achieve high-purity separation, though they are time-intensive and may cause vesicle aggregation. Size-exclusion chromatography separates EVs from soluble proteins based on hydrodynamic radius, preserving vesicle integrity and making it preferable for functional studies. Ultrafiltration uses membrane-based size cutoffs for rapid concentration, while precipitation methods offer scalability at the cost of reduced purity. Immunoaffinity isolation uses surface marker-specific antibodies to selectively capture EV subpopulations, thereby enriching the target subpopulation. Characterisation of EVs can be performed using electron microscopy or cryo-electron microscopy, depending on vesicle size and resolution requirements. Additional methods include Western blotting and immunoaffinity-based techniques to detect specific surface markers such as CD9, CD63 and CD81, as well as nanoparticle tracking analysis, which enables visualisation and measurement of vesicle size distribution based on Brownian motion

Further engineering approaches focus on modifying exosome surfaces to improve targeting efficiency. This can be achieved by incorporating targeting ligands such as peptides, aptamers, proteins, lipids, or polymers through membrane fusion or lipid–lipid interactions [99]. Techniques such as electroporation and sonication are also widely used to enhance membrane permeability and facilitate the encapsulation of proteins or RNA molecules [146]. In addition, passive loading strategies enable the incorporation of small-molecule drugs—including curcumin, paclitaxel, doxorubicin, cisplatin, and withaferin—into exosomes, thereby improving their bioavailability and therapeutic efficacy [53, 133, 157, 162, 180]. Figure 3 illustrates these exosome engineering strategies for enhancing specificity and therapeutic performance.

For microvesicles, cargo-loading strategies include electroporation, co-incubation, sonication, and endogenous loading via genetic engineering of the parent cells [131, 157]. The ARRDC1 (arrestin domain-containing protein 1) pathway represents a key endogenous mechanism for selective cargo incorporation into microvesicles. ARRDC1 recruits TSG101 to the plasma membrane, promoting outward budding and the formation of ARRDC1-mediated microvesicles (ARMMs), which can be exploited for targeted cargo delivery through genetic modification of producer cells [140].

**Fig. 4:**
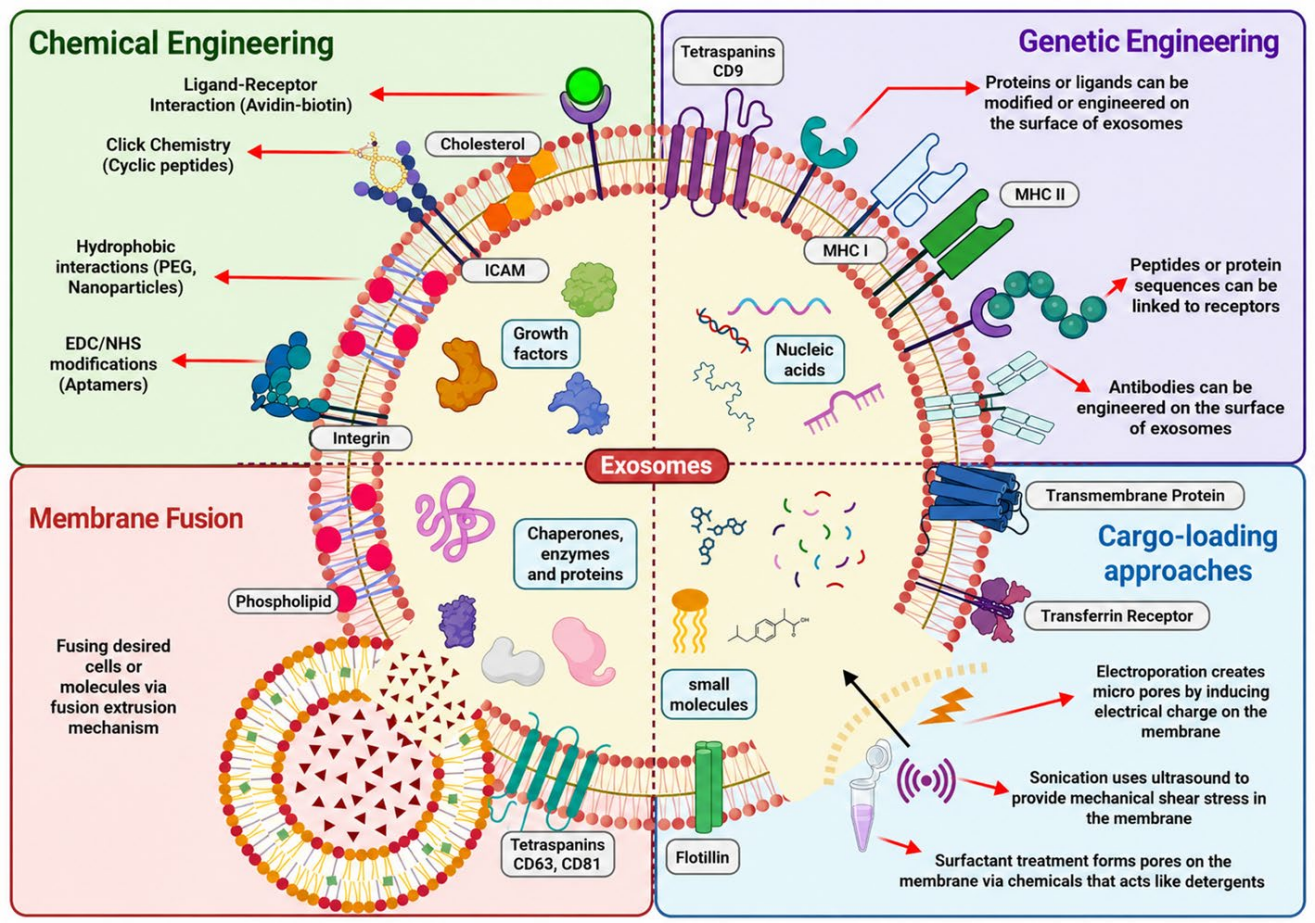
Exosome structure, composition, engineering, and cargo-loading strategies. Exosomes contain tetraspanins, cholesterol, phospholipids, transmembrane proteins, and various receptors in their plasma membrane. Engineering strategies involve three main methods: genetic, chemical and membrane fusion strategies. Genetic engineering fuses various gene, protein, or peptide sequences on the surface of the exosomes. Chemical engineering includes linking desired proteins or peptides onto exosomal receptors via various covalent and non-covalent interactions, such as biotin-avidin interactions, using cyclic peptides between the alkyne and azide groups of the receptors. EDC/NHS, in contrast, forms an amide bond between carboxyl groups and exosomal amines via aptamers. Hydrophobic reactions have been used to non-covalently modify the surface of exosomes. Membrane fusion uses an extrusion mechanism in which exosomes fuse with target membranes. Cargo can be loaded into exosomes via electroporation, sonication and surfactant treatment, which helps create micropores to load the desired molecules. *EDC/NHS- EDC (1-ethyl-3-(3-dimethylaminopropyl) carbodiimide)/NHS (N-hydroxysuccinimide); PEG- polyethylene glycol*

Targeted delivery to the CNS can be further enhanced using ligand-based strategies. For instance, the rabies virus glycoprotein (RVG) peptide binds selectively to nicotinic acetylcholine receptors expressed on neurons. Fusion of RVG with the exosomal membrane protein Lamp2b enables the generation of neuron-targeted EVs for drug delivery [76]. Notably, RVG-engineered EVs derived from immature dendritic cells have demonstrated the ability to achieve long-term gene knockdown in the CNS, supporting their potential for personalised therapeutic applications [78]. Figure 4 summarises the exosome structure, composition, and cargo-loading techniques used to engineer exosomes to increase their efficacy.

Table 2 summarises the key biomarker discoveries and therapeutic strategies targeting ALS.

**Table 2:** Summary of studies where extracellular vesicles have been used as both biomarkers and therapeutic strategies against ALS

EV Subtype	Source	Findings	References
*Biomarker studies*			
Exosomes	Plasma-derived exosomes	Exosomal TDP-43 increased at 3 and 6 months of follow-up, and plasma NfL levels were associated with rapidly progressing ALS patients	[31]
Microvesicles and exosomes	Plasma derived from sporadic ALS patients	Increased levels of SOD1, TDP-43, phospho TDP-43, and FUS in microvesicles. DOS1 was found to be more concentrated in exosomes than in microvesicles, while TDP-43 and FUS levels were higher in microvesicles than in exosomes	[154]
Exosomes	Plasma and CSF-derived exosomes	Ubiquitin-like modifying-activating protein-1, gelsolin and clusterin were found to be highly expressed in C9orf72 ALS patients	[142]
Exosomes	CSF-derived exosomes from ALS patients	Elevated levels of TDP-43	[49]
Exosomes	Plasma-astrocyte-derived exosomes	Increased levels of IL-6 correlating with ALSFRS change	[34]
Microvesicles	Plasma-leukocyte, endothelial, erythrocyte-derived microvesicles	Plasma MVs show detectable mutant SOD1 proteins	
Exosomes	CSF-derived exosomes	Increased levels of NOC2l, PDCD6IP, VCAN	
Exosomes	Plasma-derived EVs	Reduced levels of HSP90	[134]
EVs	Plasma-neural derived EVs	Elevated miR-146a-5p, miR-199a-3p, miR-151a-3p, miR-151a-5p, and miR-199a-5p, as well as decreased miR-4454, miR-10b-5p, and miR-29b-3p in plasma EVs of ALS patients	[11]
Exosomes	Plasma-derived Exosomes	positive correlation with the level of exosomal coronin-1α and ALS disease progression	[190]
EVs	CSF-derived EVs	miR-124—3p levels correlated with ALSFRS in male ALS patients	[183]
Microvesicles	Leukocyte-derived microvesicles	CD45+ MVs were elevated in ALS patients compared to healthy and AD patients	
Microvesicles	CSF-derived MVs	CD45+ MVs are elevated in the CSF of ALS patients	[12]
EVs	Astrocyte-derived EVs	GLAST+ EVs were significantly elevated in ALS patients compared to healthy controls	[143]
*Biotherapeutic studies*			
Exosomes	Adipose stem cell-derived exosomes	Exosomal miRNA21, miRNA222, and miRNAlet7a, known to prevent apoptosis and support cell proliferation, were elevated in NSC-34 motor neuron cells following exosome treatment	[20]
Exosomes	Adipose stem cell-derived exosomes	Adipose MSC-exosomes could reduce the SOD-1 aggregations and restore mitochondrial function	[98]
Exosomes	MSC-derived exosomes	The presence of ciliary neurotrophic factor in the MSC-derived exosomes facilitated motor neuron survival via the P13K/AKT pathway and promoted axonal sprouting in SOD1-G93A mice	[65]
Exosomes	Adipose stem cell-derived exosomes	Repeated intranasal administration of adipose stem-derived exosomes in SOD1-G93A mice from the onset of the disease until the end stage prevented motor neuron degeneration, reduced neuroinflammation, and preserved motor function	[21]
Exosomes	Bone marrow-endothelial cell-derived exosomes	Prevented endothelial damage induced by plasma derived from SOD1-G93A mice	[56]
Microvesicles	Bone-marrow MSC-derived MVs	Exhibited mitochondrial restorative effects, with the capacity to transfer healthy mitochondria to the defective cells	[139]
EVs	Adipose stem cell-derived EVs	Improved motor performance in ALS mice and inhibits muscle atrophy	[168]
MVs and Exosomes	MSC-derived MVs and exosomes	suppress pro-inflammatory factors while enhancing the expression of anti-inflammatory factors, thereby mitigating brain inflammation and exerting neuroprotective effects	[174]
Apoptotic bodies		dampen TLR-5/NF-KB mediated inflammatory signalling in microglia	[27]
EVs	Modified BM-MSC EVs	modified BM-MSC-derived EVs preconditioned with interferon-γ possess higher levels of miR-466q and miR-467f, which can reduce tumor necrosis factor IL-1b and prevent the activation of microglial cells	[60]
Microvesicles	Human RVG-EVs from Immature Dendritic cells	RVG-modified EVs selectively bind to nicotinic acetylcholine receptors, which are highly expressed on neurons, enabling long-term gene knockdown in the CNS, paving the way for clinical use	[78]

## Conclusion

Amyotrophic lateral sclerosis is a multifactorial neurodegenerative disease. By the time clinical symptoms manifest, substantial and often irreversible motor neuron damage has already occurred, underscoring the critical need for early diagnosis to slow disease progression. However, conventional imaging techniques can lack specificity and may lead to misdiagnosis with other motor neuron disorders. In this context, identifying reliable biomarkers, particularly those derived from EVs, offers a promising avenue for early detection and disease monitoring.

In parallel with biomarker development, therapeutic strategies for ALS are evolving. Currently, gene therapies targeting mutant genes in familial ALS are being explored using CRISPR/Cas9 genome editing and antisense oligonucleotides, aiming to directly modify or suppress pathogenic gene expression. Complementing these approaches, exosome-based therapies have emerged as a compelling strategy due to their ability to cross biological barriers and deliver functional biomolecules.

Exosomes derived from adult mesenchymal stem cells can modulate degenerating motor neurons through multiple mechanisms, including the transfer of proteins, enzymes, mRNAs, miRNAs, and siRNAs to recipient cells. These vesicles can stimulate endogenous growth factor expression, restore redox balance, exert immunomodulatory effects, and activate pro-survival signalling pathways. Furthermore, exosomes can be bioengineered to enhance their targeting specificity and, when combined with tissue-engineering approaches, hold significant potential to improve therapeutic efficacy in ALS.

## Data Availability

No datasets were generated or analysed during the current study.

## Abbreviations

AAV: Adeno-associated viral vector
ALS: Amyotrophic lateral sclerosis
AD-MSCs: Adipose-derived mesenchymal stem cells
AMSCs: Amniotic mesenchymal stem cells
AMPA: Alpha amino-3-hydroxy-5-methyl-4-isoxazole propionic acid
ARRDC1: Arrestin domain-containing protein 1
ARMMs: ARRDC1-mediated microvesicles
BBB: Blood brain barrier
BDNF: Brain-derived neurotrophic factor
BM-MSCs: Bone marrow mesenchymal stem cells
C9ORF72: Chromosome 9 open reading frame 72
CM: Conditioned media
CNS: Central nervous system
CSF: Cerebrospinal fluid
DPSCs: Dental pulp stem cells
EAAT: Excitatory amino acid transporter
EV: Extracellular vesicle
fALS: Familial amyotrophic lateral sclerosis
FTD: Frontotemporal dementia
FUS: Fused in sarcoma
GDNF: Glial derived neurotrophic factor
GOF: Gain of function
GPCs: Glial progenitor cells
IL: Interleukin
IPSC: Induced pluripotent stem cells
LMN: Lower motor neuron
LOF: Loss of function
miR: MiRNA
MSC: Mesenchymal stem cells
NMJ: Neuro-muscular junction
NSC: Neural stem cells
OECs: Olfactory ensheathing cells
OPTN: Optineurin
ROS: Reactive oxygen species
RVG: Rabies virus glycoprotein
sALS: Sporadic amyotrophic lateral sclerosis
SOD1: Superoxide dismutase 1
TARDBP: TAR-DNA binding protein
TBK-1: Tank binding kinase 1
TDP-43: TAR-DNA binding protein 43
UMN: Upper motor neuron
